# Ophthalmic nurses: meeting the need for human resources to end trachoma

**Published:** 2020-12-31

**Authors:** Amir Bedri Kello, Caleb Mpyet

**Affiliations:** 1 MD, MSc, Medical Officer, Trachoma, WHO/AFRO ESPEN, Brazzaville, Republic of Congo.; 2 Professor of Ophthalmology, University of Jos, Jos, Nigeria.

**Remote communities have fewer doctors but a greater need for trachoma services; ophthalmic nurses can fill the gap.**

**Figure:**
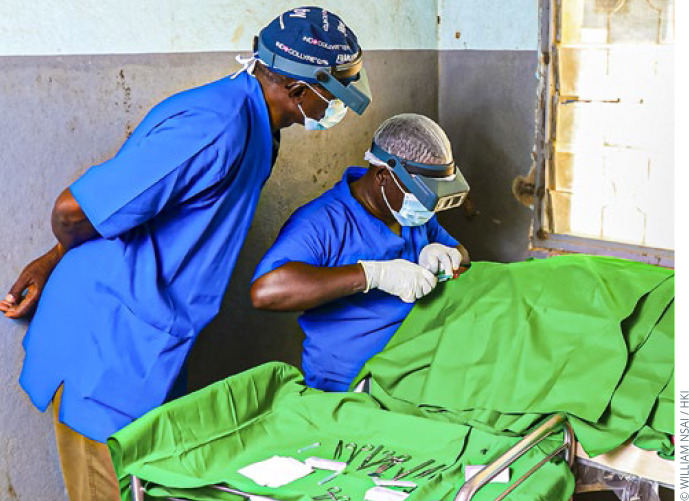
Ophthalmic nurses can be trained to carry out trachomatous trichiasis surgery. **CAMEROON**

Significant global scale-up and increased access to medical and environmental interventions in recent years have resulted in a 91% reduction in the number of people at risk of trachoma, the world's leading infectious cause of blindness: from 1.5 billion in 2002 to 142.2 million in 2019.^1^ Despite this, 2.5 million people still require urgent surgery to treat trachomatous trichiasis (TT), the late blinding stage of the disease.

Acceleration towards the elimination of trachoma as a public health problem and the achievement of universal eye health coverage is hindered because there are not enough people who are qualified to deliver comprehensive and integrated eye health interventions, including those for trachoma. The 2019 World Health Organization (WHO) World Report on Vision (**bit.ly/world-report-on-vision**) reports an inequitable distribution, and a significant shortfall, in the current and projected number of ophthalmologists, particularly in low- and middle-income countries.^2^ This also extends to an inequitable distribution between urban and rural areas.

In many sub-Saharan African countries, the shortage of ophthalmologists available to serve rural settings is being overcome by involving ophthalmic nurses, as they are trained in eye care and can provide primary eye services. In national trachoma programmes, ophthalmic nurses support disease prevalence surveys and mass drug administration campaigns, and perform eyelid surgery on people who have developed TT.

**“In many Sub-Saharan African countries, the shortage of ophthalmologists available to serve rural settings is being overcome by employing ophthalmic nurses.”**

In most sub-Saharan African countries, ophthalmic nurses are trained to carry out minor eyelid operations. Trachoma and TT mainly affect people living in remote underserved communities, where there is lack of ophthalmologists. It has been shown that trained ophthalmic nurses can perform TT surgery at the community level, close to where patients live, in a safe and effective manner.^3^

Ensuring high-quality training and supervision for health workers performing TT surgery is critical for maintaining high-quality surgical outcomes. Training includes instruction on life-like surgical mannequins, such as HEAD-START (**bit.ly/CEHJheadstart**) and operating on patients under close supervision. Health ministries are supported by several resources for training and maintaining training standards, including the World Health Organization guidelines for trichiasis surgery and final assessment of TT surgeons, titled: ‘Trichiasis surgery for trachoma’ (**bit.ly/TTsurgery**). This is accompanied by the International Coalition for Trachoma Control's preferred practices manuals, including ‘Training trichiasis surgeons for trachoma elimination programs’ (**bit.ly/TTtrain**) and ‘Supportive supervision for trachomatous trichiasis programmes’ (**bit.ly/superTT**). New tools, such as the TT tracker **bit.ly/trackTT**) enable health ministries to track the performance of health workers, including the quality of the operations performed.

The World Health Organization's World Report on Vision, and their Road Map for NTDs 2021-2030 (**bit.ly/NTDroadmap**), both emphasise the critical value of integrated cross-sectoral approaches and health systems pillars to achieve universal eye health and universal health coverage for all. In a world of limited resources, ophthalmic nurses will play a crucial role in achieving progress for eye health, including neglected tropical diseases such as trachoma. To do so effectively will require ongoing training, supervision and support. The trachoma community provides several lessons and resources that can be applied to training ophthalmic nurses in low- or middle-income countries, thereby accelerating progress towards achieving eye health for all and ensuring no one is left behind.
